# A Patient-Facing Diabetes Dashboard Embedded in a Patient Web Portal: Design Sprint and Usability Testing

**DOI:** 10.2196/humanfactors.9569

**Published:** 2018-09-24

**Authors:** William Martinez, Anthony L Threatt, S Trent Rosenbloom, Kenneth A Wallston, Gerald B Hickson, Tom A Elasy

**Affiliations:** 1 Division of General Internal Medicine and Public Health Vanderbilt University Medical Center Nashville, TN United States; 2 Health Information Technology Vanderbilt University Medical Center Nashville, TN United States; 3 Department of Medicine Vanderbilt University Medical Center Nashville, TN United States; 4 Department of Biomedical Informatics Vanderbilt University Medical Center Nashville, TN United States; 5 Department of Pediatrics Vanderbilt University Medical Center Nashville, TN United States; 6 School of Nursing Vanderbilt University Nashville, TN United States; 7 Quality, Safety & Risk Prevention Vanderbilt University Medical Center Nashville, TN United States

**Keywords:** diabetes mellitus, type 2, patient portals, qualitative research, consumer health informatics

## Abstract

**Background:**

Health apps and Web-based interventions designed for patients with diabetes offer novel and scalable approaches to engage patients and improve outcomes. However, careful attention to the design and usability of these apps and Web-based interventions is essential to reduce the barriers to engagement and maximize use.

**Objective:**

The aim of this study was to apply design sprint methodology paired with mixed-methods, task-based usability testing to design and evaluate an innovative, patient-facing diabetes dashboard embedded in an existing patient portal and integrated into an electronic health record.

**Methods:**

We applied a 5-day design sprint methodology developed by Google Ventures (Alphabet Inc, Mountain View, CA) to create our initial dashboard prototype. We identified recommended strategies from the literature for using patient-facing technologies to enhance patient activation and designed a dashboard functionality to match each strategy. We then conducted a mixed-methods, task-based usability assessment of dashboard prototypes with individual patients. Measures included validated metrics of task performance on 5 common and standardized tasks, semistructured interviews, and a validated usability satisfaction questionnaire. After each round of usability testing, we revised the dashboard prototype in response to usability findings before the next round of testing until the majority of participants successfully completed tasks, expressed high satisfaction, and identified no new usability concerns (ie, stop criterion was met).

**Results:**

The sample (N=14) comprised 5 patients in round 1, 3 patients in round 2, and 6 patients in round 3, at which point we reached our stop criterion. The participants’ mean age was 63 years (range 45-78 years), 57% (8/14) were female, and 50% (7/14) were white. Our design sprint yielded an initial patient-facing diabetes dashboard prototype that displayed and summarized 5 measures of patients’ diabetes health status (eg, hemoglobin A_1c_). The dashboard used graphics to visualize and summarize health data and reinforce understanding, incorporated motivational strategies (eg, social comparisons and gamification), and provided educational resources and secure-messaging capability. More than 80% of participants were able to successfully complete all 5 tasks using the final prototype. Interviews revealed usability concerns with design, the efficiency of use, and content and terminology, which led to improvements. Overall satisfaction (0=worst and 7=best) improved from the initial to the final prototype (mean 5.8, SD 0.4 vs mean 6.7, SD 0.5).

**Conclusions:**

Our results demonstrate the utility of the design sprint methodology paired with mixed-methods, task-based usability testing to efficiently and effectively design a patient-facing, Web-based diabetes dashboard that is satisfying for patients to use.

## Introduction

### Background

Diabetes is a leading cause of kidney failure, heart disease, stroke, visual impairment, and nontraumatic lower limb amputations [1]. Many of these complications can be delayed or prevented through disease control. Research demonstrates that diabetes self-monitoring, preventative health services, medication adherence, regular exercise, and attention to diet can lead to improved outcomes [2,3]. Despite their importance, few patients consistently receive all recommended services or engage in recommended self-care behaviors that can be challenging to implement and sustain [4,5]. Many patients with diabetes struggle with the knowledge and motivation necessary to successfully manage their disease [6].

Interventions aimed at enhancing patients’ motivation, skills, knowledge, and confidence in diabetes self-care have had limited success, with many relying on face-to-face interactions that are costly and challenging to scale [7,8]. Web-based diabetes self-management interventions have the potential to overcome these limitations; however, these interventions have also demonstrated variable effects on patients’ self-care and glycemic control [9,10]. Mixed results have been attributed to differences in the design and usability of these Web-based interventions, leading to varying degrees of user engagement [10,11]. Web-based interventions with greater user engagement are associated with better outcomes [12,13]. However, some Web-based interventions have not involved end users in the design process [14,15], and many have failed to include one or more recommended features for increasing patient engagement, including (1) ability to track, visualize, and summarize health data; (2) guidance in response to the data displayed; (3) ability to communicate with health care providers; (4) peer support; and (5) motivational challenges using elements of game design and competition [11,16].

Human-centered design is an approach to software development that emphasizes optimal user experience by integrating users directly into the design process and helps ensure the creation of a suitable user interface [17,18]. One human-centered design method, called design sprint, is a rapid 5-phase user-centered process that utilizes design principles to understand the problem, explore creative solutions, identify and map the best ideas, prototype, and ultimately test [17,18]. Usability testing ensures that Web-based interventions meet users’ expectations and work as intended, such that users are able to efficiently and effectively interact with the website [11]. Although usability testing is sometimes performed once the Web-based intervention has been fully developed, incorporating usability testing into the design process beginning with the earliest prototype provides the greatest opportunity to inform and improve the user interface design [17,18].

### Objectives

This paper describes the application of design sprint methodology paired with mixed-methods, task-based usability testing to design and evaluate an innovative, patient-facing diabetes dashboard embedded in an existing patient portal, My Health at Vanderbilt (MHAV) [19] and integrated into an electronic health record. In particular, we sought to design a dashboard that addresses the needs of users, allows users to easily comprehend their diabetes health data, incorporates recommended strategies for increasing user engagement, and is satisfying and easy to use.

## Methods

### Dashboard Design

We utilized a 5-day design sprint methodology [17,18] developed by Google Ventures (Alphabet Inc, Mountain View, CA) to design our initial dashboard prototype. The process was facilitated by an experienced health information technology expert (ALT) who specializes in user experience (UX) and product design. A 5-day design sprint approach was chosen over other iterative agile methodologies because a design sprint approach offered the ability to rapidly develop a user-centered solution in the form of a prototype that could be tested and revised before investing limited research funds into the programming of the dashboard.

**Figure 1:**
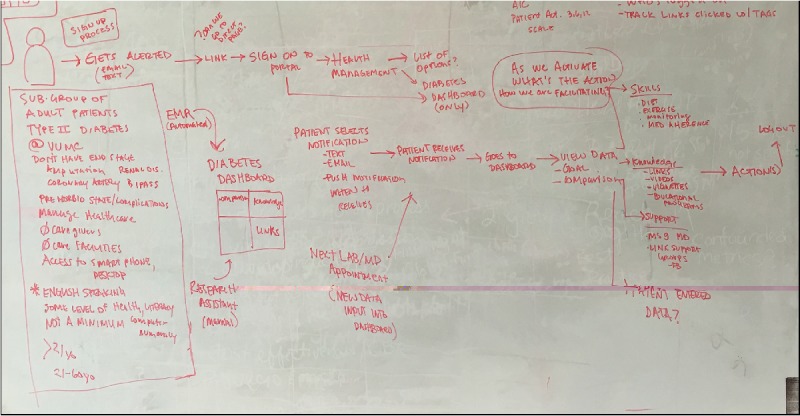
Whiteboard image mapping out challenge to create a patient-facing, diabetes dashboard.

On day 1, we began by mapping out our challenge (Figure 1) to create a dashboard that would satisfy patients’ desire for information regarding their diabetes health status and address existing challenges in patients’ diabetes knowledge and motivation for diabetes self-management [5,20]. This process was informed by a review of the literature [14,21-30] from which we identified factors contributing to the limited efficacy of existing digital interventions, including (1) absence of user-centered design [14], (2) lack of integration with the health care delivery system [22,28], (3) absence of key features to maximize patient engagement, including patient-centered motivational strategies [29], and (4) failure to account for the unique needs of older patients and those with limited health literacy [30-32]. In addition, we reviewed recommended strategies to increase patient activation [6,33] (ie, the motivation, knowledge, skills, and confidence for managing one’s health condition) using mobile apps [16] and prior research on the potential role of social comparison information for motivating diabetes self-care [27,34].

**Figure 2:**
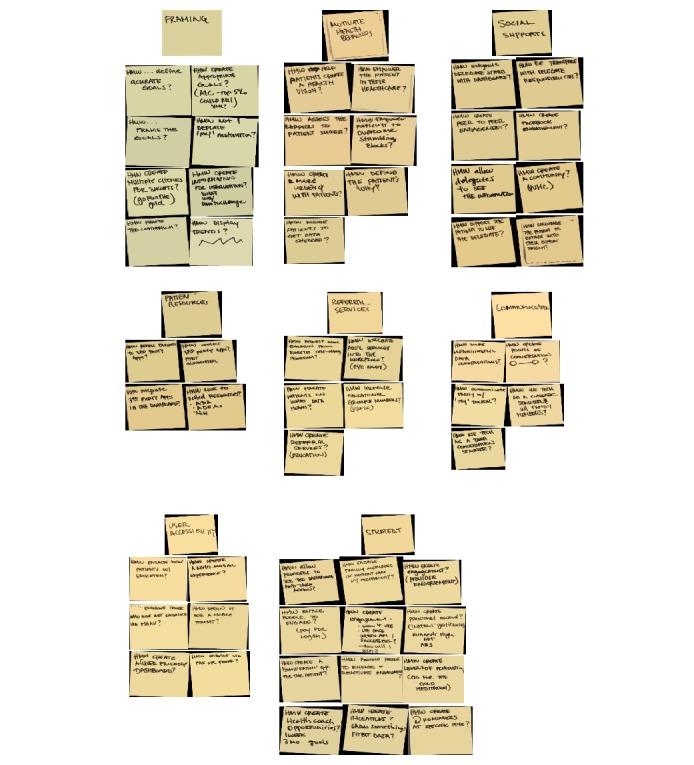
Design sprint day 1—expert comments/ideas organized into categories.

We also met one-on-one with expert stakeholders (eg, patient portal users with diabetes, diabetes educators, behavioral scientists, physicians, educators, and nurses) to ask questions aimed at enhancing our understanding of the challenge and refine our map. We identified expert stakeholders by approaching organizational leaders with a description of the project and by asking them to identify individuals in their area who could provide valuable input. For example, we approached the director of the Vanderbilt University Hospital Patient and Family Advisory Council who connected us with patients from the Council, who had diabetes, were current patient portal users, and expressed interest in improving care for people with diabetes. Experts’ comments were recorded in the form of *how might we* (HMW) statements [17,18]. The HMW method is used in design thinking to take insights and challenges and reframe them as opportunities [17,18]. Consistent with design sprint methodology, experts’ HMW statements were reviewed (Figure 2) to identify statements that shared a common theme. This was followed by grouping the statements into categories based on emerging themes to identify the most useful ideas for building the prototype. Experts encouraged the authors to consider how we might design the dashboard to (1) maximize accessibility, (2) frame diabetes health data in ways that promote patients’ understanding and motivate health behaviors, (3) facilitate patient action in response to the data they see (eg, patient resources and referral services), (4) enable communication with their health care team, (5) enhance social supports, and (6) incorporate strategies (eg, goal setting, progress tracking, and positive reinforcement) that motivate health behavior and keep users engaged.

**Figure 3:**
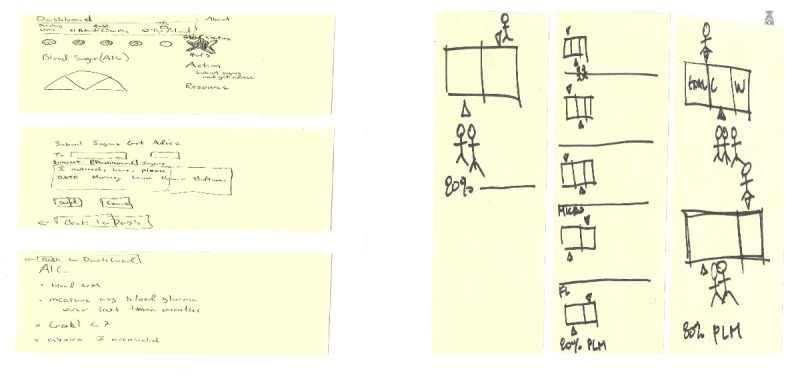
Design sprint day 2—solution sketches.

On day 2, the existing ideas, architecture, and designs from health care and other industries related to the challenge were reviewed to establish the building blocks of our prototype. For example, existing solutions for displaying health and performance data and other types of quantitative, longitudinal, and benchmarked data from other industries (eg, finance and education) were reviewed. Subsequently, findings from the review and the meetings with expert stakeholders were used to sketch our own solutions (Figure 3).

**Figure 4:**
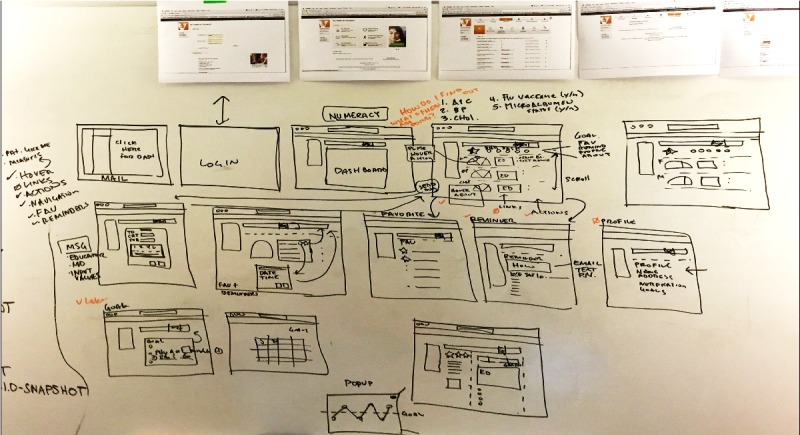
Design sprint day 3—dashboard storyboard.

On day 3, the solutions were critiqued and the solutions that had the greatest potential to successfully meet the challenge in the long term were decided by consensus. Following this, the authors adapted the solutions chosen to create a storyboard or step-by-step plan for the prototype (Figure 4).

**Figure 5:**
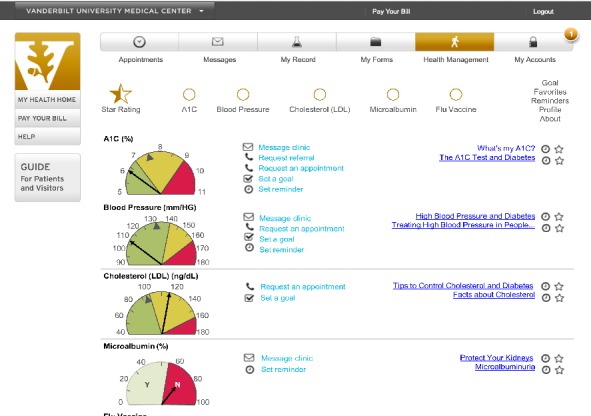
Design sprint day 4—screenshot of initial dashboard prototype. A_1c_: hemoglobin A_1c_.

On day 4, the authors developed the prototype using Apple Keynote (Apple Inc, Cupertino, CA) [35]. They collected assets (eg, stock imagery or icons) and stitched all components of the prototype together. Keynote slides (ie, screens) were tethered together using the *animate* feature to transition from one slide (ie, screen) to the next based on the action the user performs within the prototype. This resulted in an initial prototype (Figure 5) that functioned similar to a real webpage and was ready for the first round of usability testing on day 5. The initial prototype displayed and summarized 5 measures of patients’ diabetes health status (ie, hemoglobin A_1c_ [HbA_1c_], systolic blood pressure, low-density lipoprotein cholesterol, microalbumin, and flu vaccination status). The existing literature on patient’s information needs when interpreting test results and strategies for improving comprehension was reviewed [36-38]. In addition, the authors identified recommended strategies for using patient-facing technologies to increase patient activation and incorporated dashboard functionality that matched each strategy. For example, for each measure, the dashboard used graphics to visualize and summarize health data and reinforce understanding with a color-coded system (red, yellow, and green) similar to the National Heart, Lung, and Blood Institute’s asthma treatment guideline [39] to indicate when action is needed. To facilitate understanding, we paired each measure with hyperlinks to literacy level–appropriate educational materials. To help motivate patients, the dashboard provided patients with social and goal-based comparison information regarding their diabetes health status [27,34]. In addition, using elements of game design, a star rating provided patients with feedback on the number of measures at goal. To facilitate communication with their health care team, patients could click a link to contact their doctor’s office via a secure message. Reminders for self-care (eg, take medication, exercise, etc) could be set and delivered to patients’ mobile phones or email, and diabetes self-care goals could be set and tracked.

### Usability Study Design

From September to October 2016, we conducted a mixed-methods, task-based usability study of dashboard prototypes with individual patients under controlled conditions. Patients were recruited from the Vanderbilt Adult Primary Care (VAPC) clinic. Individual usability sessions lasted between 30 and 75 min. Given that the majority of usability problems are commonly identified within the first 5 usability evaluations [40-42], each round of usability testing included between 3 and 6 participants. After each round of usability testing, the dashboard prototype was revised in response to usability findings before the next round of testing.

### Setting

The VAPC clinic is located within the Vanderbilt University Medical Center (VUMC) in Nashville, TN. The clinic cares for about 25,000 unique patients annually, of which about 4500 (18.00%) have diabetes. All clinical data are entered into an electronic health record, and the patients are provided access to their clinical data via a Web portal.

### Participants and Recruitment

Participants were eligible for the study if they had type 2 diabetes mellitus, were English-speaking, were aged 21 years or older, and were current users of the VUMC patient Web portal, MHAV. Potential participants were identified automatically using VUMC’s Subject Locator to query the electronic health records of patients with upcoming clinic appointments for discrete inclusion and exclusion criteria. Identified patients (n=334) were mailed a letter describing the study and asked to contact the investigators if they were interested in participating. Interested patients (n=22) contacted the research coordinator to learn more about the study and confirm eligibility. Patients who agreed to participate (n=17) were scheduled to participate in a usability session on the day of their clinic appointment. Overall, 3 patients canceled due to weather or a conflicting appointment. A total of 14 patients ultimately completed a usability session and provided written informed consent before participating in their session. The Vanderbilt University Institutional Review Board approved this research.

### Data Collection and Measures

Before the usability testing session, enrolled patients were asked to complete a short questionnaire before their interview. The questionnaire included basic demographic questions, including items about computer and smartphone usage and internet access, as well as validated measures of health literacy [43] and numeracy [44]. In addition, data regarding comorbidities were extracted from participants’ medical record as reported by the physicians within the patients’ problem list.

Each participant received a standardized introduction to the dashboard and the *think-aloud* procedure that allows testing observers to understand and track a participant’s thought processes as they navigate the dashboard [45]. One of the authors (ALT) led each session using a semistructured interview guide, while another author (WM) observed and took notes. With a dashboard prototype that contained fictitious patient data, participants were asked to perform common standardized tasks including logging in, retrieving HbA_1c_ data, messaging their doctor, setting a reminder, and setting a goal. The tasks were designed to represent what typical users might do when visiting their dashboard. All participants accessed and navigated the dashboard using a 15-inch MacBook Pro 11,3 (2014 generation) with an external mouse and Chrome Web browser with default resolution. In addition, after participants attempted each assigned task (eg, message your doctor), the interviewers used open-ended questions outlined in the interview guide to elicit participants’ (1) expectations for the feature’s functionality, (2) ability to comprehend the information displayed, (3) ability to navigate to and from the feature, (4) satisfaction with the feature, and (5) how the feature might be improved. Each session was audio-recorded, and the computer screen was video-recorded using QuickTime Player (Apple Inc, Cupertino, CA).

To assess and quantify participant satisfaction with the dashboard, at the conclusion of their usability session, participants completed 12 items from the Computer System Usability Questionnaire (CSUQ), which assess participants’ perceptions of the dashboard’s ease of use, likability of the interface, and overall satisfaction using a 7-point Likert response scale (1=strongly disagree to 7=strongly agree), with 7 indicating the highest possible satisfaction [46].

### Data Analysis

#### Task Completion Analysis

Task completion was coded with a usability rating scale utilized in prior studies [47-49]. Task completion was rated on a 5-category scale: (1) successful/straightforward, (2) successful/prolonged, (3) partial, (4) unsuccessful/prolonged, and (5) gave up [47]. Two coders first coded the same usability session video (not used in the analysis) to calibrate their coding. They subsequently coded the remaining videos independently. Disagreements were resolved by consensus, and both coders were blinded to the dashboard prototype representing the initial prototype and the prototypes that were revisions.

#### Interview Analysis

Audio files of interviews were submitted to a professional transcription service, Rev.com Inc (San Francisco, CA). Transcripts were checked for accuracy and identifying information was removed. Deidentified transcripts were imported into NVivo 10 (version 10; QSR International, Burlington, VT) for coding and analysis. Similar to other health app usability studies [47,50], we used selective coding to capture participants’ comments about usability concerns [51]. Participant comments were sorted into categories that addressed 3 elements of usability: design, efficiency of use, and content and terminology [52]. A research assistant with training in qualitative methods coded all interviews. After the initial coding, a second trained coder reviewed each code and noted any discrepancies. The 2 coders then met and resolved any differences by consensus. Illustrative quotes from participants were edited slightly for grammar and clarity for inclusion in this paper. Participants’ comments informed revisions to the dashboard prototype.

### Statistical Analysis

Descriptive statistics were used to characterize the study participants, task completion, and survey data. All analyses were completed with SAS version 9.4 (SAS Institute, Inc, Cary, NC).

### Stop Criteria

Data analysis began after the initial round of testing, and the authors used the findings to inform prototype revisions before the subsequent round of testing. Additional rounds of testing were conducted until the majority of participants within a round of testing (1) were able to successfully complete all tasks, (2) indicated high overall satisfaction with the dashboard as assessed by the overall satisfaction item on the CSUQ (score≥6), and (3) expressed no new usability concerns during the interview (ie, saturation).

## Results

### Participants

Table 1 shows participant characteristics. The sample (N=14) comprised 5 patients in round 1, 3 patients in round 2, and 6 patients in round 3; at this point, the authors reached their stop criteria. Participants’ mean age was 63 years (range 45-78 years), 57% (8/14) were female, and 50% (7/14) were white. All participants reported using a home computer, and 64% (9/14) reported using a smartphone. All participants had home internet access. Most participants had one or more comorbid diseases in addition to diabetes.

### Task-Based Usability

**Figure 6:**
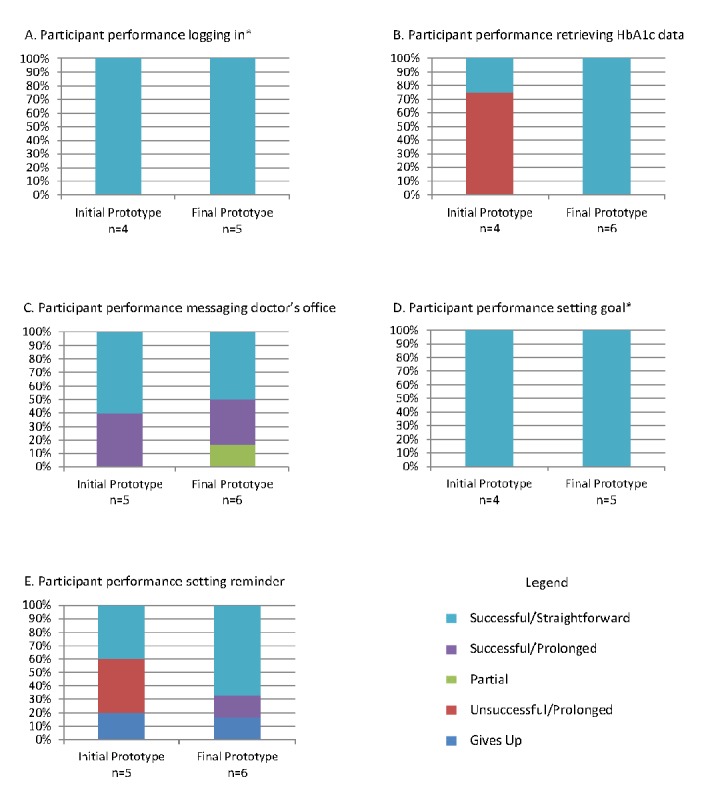
Task-based usability ratings for initial and final prototype iterations. The asterisk indicates that one participant within the final round of testing was not asked to complete the task due to time constraints. HbA1c: hemoglobin A1c.

Figure 6 illustrates task performance among the 5 participants in round 1 who tested the initial prototype compared with the 6 participants in round 3 who tested the final prototype. Participants attempted 5 tasks that ranged in complexity from logging in to setting a reminder.

#### Tasks: (A) Log-In and (B) Set a Goal

All participants in both rounds straightforwardly logged in to the dashboard and set a goal.

#### Task: (C) Identify Most Recent Hemoglobin A _1c_

**Figure 7:**
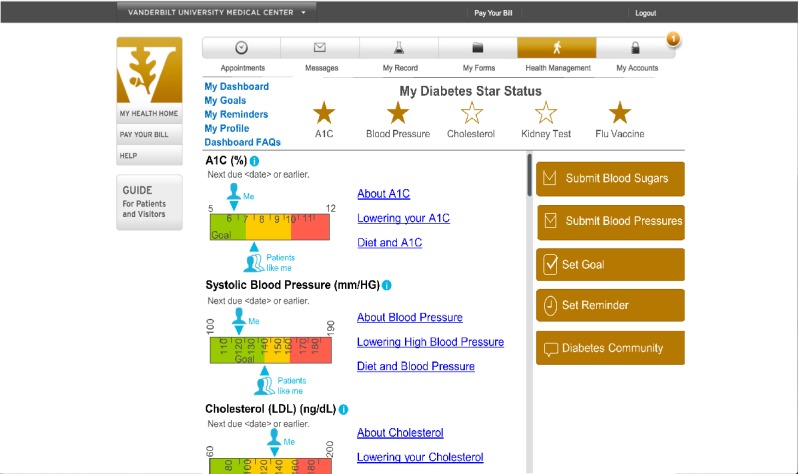
Screenshot of final dashboard prototype. A_1c_: hemoglobin A_1c_.

Only one participant in the initial round of testing was able to identify their most recent HbA_1c_ value from the dashboard. Most participants had difficulty interpreting the dial display, were confused regarding which icon on the dial indicated the user’s most current value, and could not comprehend the HbA_1c_ data. In response, the authors revised the data display design and status indicator icons. They relocated the features aimed at facilitating patients’ understanding of their health data, including a hover over info icon providing a nontechnical description of the measure (eg, HbA_1c_) and links to literacy level–sensitive educational materials so they were adjacent to the data (see Figure 1 initial prototype and Figure 7 final prototype). After revisions, all 6 participants in the final round were able to complete the task and comprehend their data.

#### Task: (D) Message Doctor’s Office

All 5 participants in the initial round were able to message their doctor’s office; however, 2 participants hesitated or demonstrated some confusion despite completing the task. Participants indicated that they were accustomed to using the existing messaging icon within the header of the patient portal, and some struggled to locate the messaging icon within the dashboard. After revising the icon in response to feedback (ie, larger text, adding color and a button icon), the majority of participants in the final round successfully completed the task. However, 3 participants continued to initially attempt messaging via the existing icon in the header, one of whom completed the task only after being directed to the correct button icon.

**Table 1:** Participant characteristics.

Characteristic		Total (N=14)	Round 1 (N=5)	Round 2 (N=3)	Round 3 (N=6)
Age (years), mean (SD)		63.4 (11.0)	62.2 (10.3)	75.7 (3.2)	58.2 (9.9)
**Age (years), n (%)**					
	40-49	1 (7)	0 (0)	0 (0)	1 (16)
	50-59	4 (29)	2 (40)	0 (0)	2 (33)
	60-69	4 (29)	2 (40)	0 (0)	2 (33)
	70-79	5 (36)	1 (20)	3 (100)	1 (16)
**Gender, n (%)**					
	Female	8 (57)	3 (60)	0 (0)	5 (83)
	Male	6 (43)	2 (40)	3 (100)	1 (17)
**Race, n (%)**					
	White	7 (50)	3 (60)	1 (33)	3 (50)
	African American	3 (21)	1 (20)	1 (33)	1 (17)
	Asian	2 (14)	1 (20)	1 (33)	0 (0)
	Other	2 (14)	0 (0)	0 (0)	2 (33)
**Education**, **n (%)**					
	High school degree / graduate equivalency degree	1 (7)	1 (20)	0 (0)	0 (0)
	Some college	3 (21)	1 (20)	0 (0)	2 (33)
	College degree	5 (36)	1 (20)	2 (67)	2 (33)
	Postgraduate degree	5 (36)	2 (40)	1 (33)	2 (33)
Health literacy, mean (range^a^)		13.4 (11-15)	13.2 (12-15)	12.7 (11-15)	14.0 (13-15)
Numeracy, mean (range^b^)		15.0 (7-18)	13.0 (7-18)	17.0 (16-18)	15.7 (10-18)
Home computer user^c^, n (%)		14 (100)	5 (100)	3 (100)	6 (100)
Smartphone user, n (%)		9 (64)	3 (60)	2 (67)	4 (67)
Home internet access, n (%)		14 (100)	5 (100)	3 (100)	6 (100)
**Comorbidities, n (%)**					
	Hyperlipidemia	10 (71)	3 (60)	3 (100)	4 (67)
	Atherosclerotic cardiovascular disease	3 (21)	0 (0)	1 (33)	2 (33)
	Hypertension	7 (50)	2 (40)	3 (100)	2 (33)
	Chronic kidney disease	3 (21)	1 (20)	1 (33)	1 (17)

^a^Possible score range: 3 (worst) to 15 (best).

^b^Possible score range: 3 (worst) to 18 (best).

^c^Includes desktops, laptops, or tablets.

#### Task: (E) Set a Reminder

Only 2 participants in round 1 were able to set a reminder on the dashboard. Participants struggled to set the frequency of recurrence and a stop date for reminders they wished to receive only for a specified time. Subsequently, the authors revised the layout of the “set reminder” pop up window to include a clear start and stop date and time, as well as a drop-down menu to set recurrences (eg, daily, weekly, etc). After revisions, 4 of 6 participants in round 3 were able to set a reminder, with one additional participant successfully completing the task with prolonged effort.

### Participant Interviews

Table 2 shows the participants’ comments about usability concerns grouped by usability area. Several revisions were made in response to participants’ usability concerns, including revisions to the display of patients’ health data and star status, icons indicating the patient’s value and “patients like me” value, standardizing educational links and adding diet information, grouping and standardizing action items, enlarging the font size, and providing a frequently asked questions page (see Figure 1 initial prototype and Figure 7 final prototype).

### Satisfaction Survey

Table 3 reports mean scores for the CSUQ items among participants in round 1 who tested the initial prototype compared with participants in round 3 who tested the final prototype. Participants who tested the initial prototype and those who tested the final prototype rated the usability above average (ie, scores >4 on a 7-point scale) for all 12 items. The mean score for all 12 items improved between the initial and final prototypes.

**Table 2:** Participants’ concerns with dashboard usability.

Usability element and unique concern type			Illustrative quote
**Design**			
	Font size	*It’s very clear to me but I would definitely want to enlarge the size of the font.*	
	Patient status indicator	*I don’t know what [the indicator] is supposed to be. Still I want to figure it out. The person’s goal would be about 6.2 and the actual would be 7.5. Is that correct?*	
	Reminder functionality	*That’s a reminder, oh! That’s a clock symbol. Gotcha. It could be clearer [laughs].*	
	Patients like me indicator	*Not clear that this [icon] is for individual. This [icon] is for group. Up to here [group icon], just add one more figure so that will show more people.*	
	Star rating	*There’s a star over here, on this side, but does it indicate the same thing as the star rating over here? By rating, is that telling me that I’m doing poor, good, with my goals?*	
	Hover over functionality	*No I wouldn’t have known [I could hover over]. Once you clicked, then I realized.*	
	Goal setting functionality	*The end date [for the goal], you’re talking about the last day of your, I don’t get that. The end date [for the goal]. Help me.*	
**Efficiency of use**			
	Redundancy	*I mean those two things [my medical concerns drop down menu] and the message subject [free text; are the same].*	
**Content and terminology**			
	Historical values	*I’d actually like to see what my last three [HbA_1c_] were.*	
	Medical jargon	*I don’t even know what [microalbumin] is. I’ve never heard of that.*	
	Diet information	*If you could just do something about diet. I don’t see that on there anywhere. I mean, because that’s like a big part of it, like what can I eat, what should I eat.*	
	Online community	*You’re not going to be able to communicate with other patients and talk about the key things they do for support. That might be something you would add.*	

**Table 3:** Computer system usability questionnaire survey items assessing the dashboard usability: initial versus final prototype.

Item	Initial prototype (n=5), mean (SD)	Final prototype (n=6), mean (SD)
Overall, I am satisfied with how easy it is to use this system.	5.6 (1.1)	6.3 (0.8)
It is simple to use this system.	6.0 (0.8)	6.3 (0.8)
I feel comfortable using this system.	5.7 (1.3)	6.5 (1.3)
It was easy to learn to use this system.	6.2 (0.8)	6.5 (0.8)
It is easy to find the information I need.	5.6 (1.5)	4.8 (1.2)
The information provided with the system is easy to understand.	5.4 (1.7)	5.8 (1.2)
The organization of information on the system screens is clear.	4.2 (2.2)	6.5 (0.5)
The interface of this system is pleasant.	5.4 (1.3)	6.5 (0.5)
I like using the interface of this system.	5.4 (1.1)	6.5 (0.5)
The system has all the functions and capabilities I expect it to have.	6.0 (0.7)	6.2 (0.8)
Overall, I am satisfied with this system.	5.8 (0.4)	6.7 (0.5)
The system is visually appealing.	5.8 (1.3)	6.5 (0.5)

## Discussion

### Principal Findings

**Figure 8:**
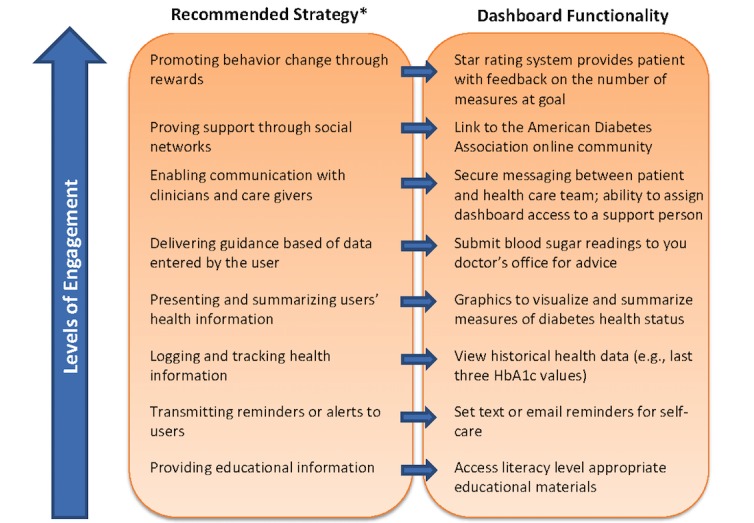
Recommended strategies for patient activation and paired dashboard functionality by level of patient engagement.The asterisk refers to the engagement pyramid reported by Singh et al, 2016 [16]. HbA_1c_: hemoglobin A_1c_.

Our study illustrates the use of design sprint methodology alongside mixed-methods, task-based usability testing in the design of a Web-based intervention for patients with diabetes. By using this design approach, we were able to rapidly create a prototype and rigorously assess task-based usability before any programming. Task-based usability testing and qualitative analysis of interviews with a small number of participants quickly identified usability challenges that led to improvements in successive iterations. Participant feedback informed changes in the data display that led to improved comprehension of diabetes health data. Participants’ usability satisfaction surveys demonstrated a high level of satisfaction with the dashboard that improved from initial to final prototype. The final prototype incorporated recommended strategies to enhance patient activation across the engagement spectrum, from providing educational resources to promoting behavior change through rewards (see Figure 8) [16].

### Building Upon Prior Research

Several prior studies have reported the design and usability of patient-facing health apps and Web-based interventions for patients with diabetes [50,53-58]. Approaches to the design of these health apps and Web-based interventions typically employ some variation of user-centered design [56-59]. A significant limitation of prior design approaches is the time and cost involved with the rapidly evolving pace of technology [60,61]. This study is the first in our knowledge to report the design of a digital health intervention using design sprint methodology and demonstrate its utility in efficiently and effectively designing a Web-based intervention that is satisfying to use.

By utilizing design sprint methodology, we were able to create a viable initial prototype within 5 days. Given the rapidly evolving technology and patient expectations of health technology [60,62], efficient yet rigorous design methodology is essential. We were able to enhance the scientific rigor of the design sprint approach by using validated measures of usability [46] and task-performance [47-49], as well as an established qualitative methodology to analyze interviews and determine saturation [51]. This approach allows usability concerns to be identified before programming, potentially saving the researcher both time and money. Consistent with the findings of Nielsen, we found that the majority of usability problems were identified in the first 5 usability evaluations, with diminishing returns after the eighth evaluation [40-42]. While enrolling additional participants in our study may have revealed additional usability concerns, our sample was sufficient to establish a minimally viable product (eg, final prototype) that allowed us to proceed to program the dashboard with the reasonable confidence that most usability issues were identified and addressed. As with any app or website, ongoing attention to user feedback and iterative improvements are likely to continue indefinitely as technology and users evolve. Although some usability studies employ a large number of participants, this is mostly done to provide sufficient sample size for quantitative analyses, and additional participants yield relatively few new usability concerns [40-42]. In addition, our usability findings build upon other recent studies of patient-facing diabetes health apps [50,53,59]. Georgsson et al used a similar mixed-methods approach to evaluate the usability of their mHealth system for diabetes type 2 self-management [53]. Similar to this study, their study included task-based testing with a *think-aloud* protocol, semistructured interviews, and a questionnaire on patients’ experiences using their system. Consistent with Georgsson et al, we found a mixed-methods approach resulted in a comprehensive understanding of usability. Our study extends these findings by demonstrating the effectiveness of this approach to objectively assess and track usability in response to iterative revisions of a prototype in the design phase.

Our study also has implications for the design of patient portals and the display of patients’ health data. By giving patients direct access to their health data, patient portals can improve patient engagement [63] and empower patients to actively participate in their care [64]. However, research suggests that patients struggle to understand health data communicated to them via patient portals [65]. A recent study by Giardian et al suggests that current patient portals do not display health data in a patient-centered way, which can lead to misunderstandings and patient distress [66]. In our study, patients had difficulty comprehending HbA_1c_ data in the dial display (Figure 1) that improved with ruler display (Figure 7), demonstrating the importance of user-centered design. Although the content was relatively unchanged, we revised the display based on user feedback, resulting in increased comprehension and improved visibility of features aimed at facilitating patients’ understanding of their health data.

### Limitations

This study has important limitations. We recruited a convenience sample of patients from a single, large, urban academic medical center that may limit the generalizability of our findings. Our sample included patients who were more educated and had greater computer and internet access than the overall population of patients with diabetes [67,68]. For future studies, researchers should consider purposive sampling to recruit patients with specific characteristics. Given the known barriers to usability among older patients [15], a strength of our sample was the inclusion of a majority of patients over the age of 60 years that allowed us to ensure the dashboard usability among this demographic. In addition, although we were able to directly observe individual users as they attempted several assigned tasks using the dashboard, our data are subject to the Hawthorne effect (ie, altered behavior due to an awareness of being observed). Similarly, we did not collect data on how patients would engage with the dashboard on their own. It would be useful to collect *actual-use* data in future studies including the level of engagement with specific dashboard functions over time. Although we designed the dashboard with elements aimed at increasing patient activation, this study focused on the design and task-based usability of the dashboard and not on the evaluation of its impact. Further research is needed to test the efficacy of the dashboard on cognitive, behavioral, and clinical outcomes including patient activation.

Researchers and others considering using design sprint methodology should also consider some of the limitations of the approach. Although a standard design sprint that unfolds over 5 days is generally recommended [17,18], researchers may wish to experiment with shorter, or more likely, longer sprints. Design sprint methodology relies on understanding the user (ie, the consumer and their needs), and in some instances, it may be necessary to spend additional time before the design sprint to understand the target user and their needs and challenges. In our case, a literature review on the patients’ experiences with portal use, challenges with diabetes self-management, and the limitations of existing diabetes apps provided insights about our target users. Design sprints also rely heavily on the ideas generated from the solutions sketched by team members on day 2. Therefore, this phase of idea generation should not be shortened and may, in fact, benefit from more time.

### Conclusions

In conclusion, the results underscore the value of design sprint methodology to efficiently create a viable user-centric prototype of a Web-based intervention and the importance of mixed-methods evaluation of usability as a part of the design phase beginning with the initial prototype. Design sprints offer an efficient way to define the problem, assess the needs of users, iteratively generate ideas and develop a viable product for testing, whereas usability evaluation methods ensure health apps and Web-based interventions appeal to users and support their use.

## Abbreviations

CSUQ: Computer System Usability Questionnaire
HbA_1c_: hemoglobin A1c
HMW: how might we
MHAV: My Health at Vanderbilt
UX: user experience
VAPC: Vanderbilt Adult Primary Care
VUMC: Vanderbilt University Medical Center

## References

[1] (2017). Centers for Disease Control and Prevention.

[2] Powers MA, Bardsley J, Cypress M, Duker P, Funnell MM, Fischl AH, Maryniuk MD, Siminerio L, Vivian E (2017). Diabetes self-management education and support in type 2 diabetes. Diabetes Educ.

[3] Shrivastava SR, Shrivastava PS, Ramasamy J (2013). Role of self-care in management of diabetes mellitus. J Diabetes Metab Disord.

[4] Bennett KJ, McDermott S, Mann JR, Hardin J (2017). Receipt of recommended services among patients with selected disabling conditions and diabetes. Disabil Health J.

[5] McBrien KA, Naugler C, Ivers N, Weaver RG, Campbell D, Desveaux L, Hemmelgarn BR, Edwards AL, Saad N, Nicholas D, Manns BJ (2017). Barriers to care in patients with diabetes and poor glycemic control: a cross-sectional survey. PLoS One.

[6] Sacks RM, Greene J, Hibbard J, Overton V, Parrotta CD (2017). Does patient activation predict the course of type 2 diabetes? A longitudinal study. Patient Educ Couns.

[7] Bolen SD, Chandar A, Falck-Ytter C, Tyler C, Perzynski AT, Gertz AM, Sage P, Lewis S, Cobabe M, Ye Y, Menegay M, Windish DM (2014). Effectiveness and safety of patient activation interventions for adults with type 2 diabetes: systematic review, meta-analysis, and meta-regression. J Gen Intern Med.

[8] Sullivan SD, Dalal MR, Burke JP (2013). The impact of diabetes counseling and education: clinical and cost outcomes from a large population of US managed care patients with type 2 diabetes. Diabetes Educ.

[9] Wu Y, Yao X, Vespasiani G, Nicolucci A, Dong Y, Kwong J, Li L, Sun X, Tian H, Li S (2017). Mobile app-based interventions to support diabetes self-management: a systematic review of randomized controlled trials to identify functions associated with glycemic efficacy. JMIR Mhealth Uhealth.

[10] Cotter AP, Durant N, Agne AA, Cherrington AL (2014). Internet interventions to support lifestyle modification for diabetes management: a systematic review of the evidence. J Diabetes Complications.

[11] Yu CH, Bahniwal R, Laupacis A, Leung E, Orr MS, Straus SE (2012). Systematic review and evaluation of web-accessible tools for management of diabetes and related cardiovascular risk factors by patients and healthcare providers. J Am Med Inform Assoc.

[12] Glasgow RE, Christiansen SM, Kurz D, King DK, Woolley T, Faber AJ, Estabrooks PA, Strycker L, Toobert D, Dickman J (2011). Engagement in a diabetes self-management website: usage patterns and generalizability of program use. J Med Internet Res.

[13] Bennett GG, Herring SJ, Puleo E, Stein EK, Emmons KM, Gillman MW (2010). Web-based weight loss in primary care: a randomized controlled trial. Obesity (Silver Spring).

[14] McCurdie T, Taneva S, Casselman M, Yeung M, McDaniel C, Ho W, Cafazzo J (2012). mHealth consumer apps: the case for user-centered design. Biomed Instrum Technol.

[15] Isaković M, Sedlar U, Volk M, Bešter J (2016). Usability pitfalls of diabetes mHealth apps for the elderly. J Diabetes Res.

[16] Singh K, Drouin K, Newmark LP, Rozenblum R, Lee J, Landman A, Pabo E, Klinger EV, Bates DW (2016). Developing a framework for evaluating the patient engagement, quality, and safety of mobile health applications. Issue Brief (Commonw Fund).

[17] Banfield R, Lombardo CT, Wax T (2015). Design Sprint: A Practical Guidebook for Building Great Digital Products.

[18] Knapp J, Zeratsky J, Kowitz B (2016). Sprint: How to Solve Big Problems and Test New Ideas in Just Five Days.

[19] Osborn CY, Rosenbloom ST, Stenner SP, Anders S, Muse S, Johnson KB, Jirjis J, Jackson GP (2011). MyHealthAtVanderbilt: policies and procedures governing patient portal functionality. J Am Med Inform Assoc.

[20] Sweileh WM, Zyoud SH, Abu Nab'a RJ, Deleq MI, Enaia MI, Nassar SM, Al-Jabi SW (2014). Influence of patients' disease knowledge and beliefs about medicines on medication adherence: findings from a cross-sectional survey among patients with type 2 diabetes mellitus in Palestine. BMC Public Health.

[21] Amante DJ, Hogan TP, Pagoto SL, English TM (2014). A systematic review of electronic portal usage among patients with diabetes. Diabetes Technol Ther.

[22] Eng DS, Lee JM (2013). The promise and peril of mobile health applications for diabetes and endocrinology. Pediatr Diabetes.

[23] Kuo A, Dang S (2016). Secure messaging in electronic health records and its impact on diabetes clinical outcomes: a systematic review. Telemed J E Health.

[24] Osborn CY, Mayberry LS, Mulvaney SA, Hess R (2010). Patient web portals to improve diabetes outcomes: a systematic review. Curr Diab Rep.

[25] Payne HE, Lister C, West JH, Bernhardt JM (2015). Behavioral functionality of mobile apps in health interventions: a systematic review of the literature. JMIR Mhealth Uhealth.

[26] Martínez-Pérez B, de la Torre-Díez I, López-Coronado M (2013). Mobile health applications for the most prevalent conditions by the World Health Organization: review and analysis. J Med Internet Res.

[27] Schokker MC, Keers JC, Bouma J, Links TP, Sanderman R, Wolffenbuttel BH, Hagedoorn M (2010). The impact of social comparison information on motivation in patients with diabetes as a function of regulatory focus and self-efficacy. Health Psychol.

[28] El-Gayar O, Timsina P, Nawar N, Eid W (2013). Mobile applications for diabetes self-management: status and potential. J Diabetes Sci Technol.

[29] Hood M, Wilson R, Corsica J, Bradley L, Chirinos D, Vivo A (2016). What do we know about mobile applications for diabetes self-management? A review of reviews. J Behav Med.

[30] Lyles CR, Sarkar U, Osborn CY (2014). Getting a technology-based diabetes intervention ready for prime time: a review of usability testing studies. Curr Diab Rep.

[31] Arnhold M, Quade M, Kirch W (2014). Mobile applications for diabetics: a systematic review and expert-based usability evaluation considering the special requirements of diabetes patients age 50 years or older. J Med Internet Res.

[32] Sarkar U, Karter AJ, Liu JY, Adler NE, Nguyen R, Lopez A, Schillinger D (2010). The literacy divide: health literacy and the use of an internet-based patient portal in an integrated health system-results from the diabetes study of northern California (DISTANCE). J Health Commun.

[33] Hibbard JH, Stockard J, Mahoney ER, Tusler M (2004). Development of the Patient Activation Measure (PAM): conceptualizing and measuring activation in patients and consumers. Health Serv Res.

[34] Martinez W, Wallston KA, Schlundt DG, Hickson GB, Bonnet KR, Trochez RJ, Elasy TA (2018). Patients' perspectives on social and goal-based comparisons regarding their diabetes health status. Br Med J Open Diabetes Res Care.

[35] Siddiqui H (2015). Keynote Animation – How To Prototype UI.

[36] Elder NC, Barney K (2012). “But what does it mean for me?” Primary care patients' communication preferences for test results notification. Jt Comm J Qual Patient Saf.

[37] Torsvik T, Lillebo B, Mikkelsen G (2013). Presentation of clinical laboratory results: an experimental comparison of four visualization techniques. J Am Med Inform Assoc.

[38] Zikmund-Fisher BJ, Scherer AM, Witteman HO, Solomon JB, Exe NL, Tarini BA, Fagerlin A (2017). Graphics help patients distinguish between urgent and non-urgent deviations in laboratory test results. J Am Med Inform Assoc.

[39] (2007). National Institutes of Health.

[40] Nielsen J, Landauer TK (1993). A mathematical model of the finding of usability problems.

[41] Nielsen J (1994). Usability inspection methods.

[42] Nielsen J (2000). Nielsen Norman Group.

[43] Sarkar U, Schillinger D, López A, Sudore R (2011). Validation of self-reported health literacy questions among diverse English and Spanish-speaking populations. J Gen Intern Med.

[44] Fagerlin A, Zikmund-Fisher BJ, Ubel PA, Jankovic A, Derry HA, Smith DM (2007). Measuring numeracy without a math test: development of the Subjective Numeracy Scale. Med Decis Making.

[45] Jaspers MW, Steen T, van den Bos C, Geenen M (2004). The think aloud method: a guide to user interface design. Int J Med Inform.

[46] Lewis JR (1995). IBM computer usability satisfaction questionnaires: psychometric evaluation and instructions for use. Int J Hum-Comput Interact.

[47] Sarkar U, Gourley GI, Lyles CR, Tieu L, Clarity C, Newmark L, Singh K, Bates DW (2016). Usability of commercially available mobile applications for diverse patients. J Gen Intern Med.

[48] Taha J, Sharit J, Czaja SJ (2014). The impact of numeracy ability and technology skills on older adults' performance of health management tasks using a patient portal. J Appl Gerontol.

[49] Segall N, Saville JG, L'Engle P, Carlson B, Wright MC, Schulman K, Tcheng JE (2011). Usability evaluation of a personal health record. AMIA Annu Symp Proc.

[50] Nelson LA, Bethune MC, Lagotte AE, Osborn CY (2016). The usability of diabetes MAP: a web-delivered intervention for improving medication adherence. JMIR Hum Factors.

[51] Strauss A, Corbin J (1990). Basics of Qualitative Research: Techniques and Procedures for Developing Grounded Theory.

[52] US Department of Health Human Services (2014). Usability.gov.

[53] Georgsson M, Staggers N (2016). An evaluation of patients' experienced usability of a diabetes mHealth system using a multi-method approach. J Biomed Inform.

[54] Georgsson M, Staggers N (2016). Quantifying usability: an evaluation of a diabetes mHealth system on effectiveness, efficiency, and satisfaction metrics with associated user characteristics. J Am Med Inform Assoc.

[55] Fu H, McMahon SK, Gross CR, Adam TJ, Wyman JF (2017). Usability and clinical efficacy of diabetes mobile applications for adults with type 2 diabetes: a systematic review. Diabetes Res Clin Pract.

[56] Grant RW, Wald JS, Poon EG, Schnipper JL, Gandhi TK, Volk LA, Middleton B (2006). Design and implementation of a web-based patient portal linked to an ambulatory care electronic health record: patient gateway for diabetes collaborative care. Diabetes Technol Ther.

[57] Osborn CY, Mulvaney SA (2013). Development and feasibility of a text messaging and interactive voice response intervention for low-income, diverse adults with type 2 diabetes mellitus. J Diabetes Sci Technol.

[58] Yu CH, Parsons JA, Hall S, Newton D, Jovicic A, Lottridge D, Shah BR, Straus SE (2014). User-centered design of a web-based self-management site for individuals with type 2 diabetes-providing a sense of control and community. BMC Med Inform Decis Mak.

[59] Alanzi T, Istepanian R, Philip N (2016). Design and usability evaluation of social mobile diabetes management system in the gulf region. JMIR Res Protoc.

[60] Patrick K, Hekler EB, Estrin D, Mohr DC, Riper H, Crane D, Godino J, Riley WT (2016). The pace of technologic change: implications for digital health behavior intervention research. Am J Prev Med.

[61] Da Silva TS, Martin A, Maurer F, Silveira M (2011). User-centered design and agile methods: a systematic review.

[62] Lithgow K, Edwards A, Rabi D (2017). Smartphone app use for diabetes management: evaluating patient perspectives. JMIR Diabetes.

[63] Tulu B, Trudel J, Strong DM, Johnson SA, Sundaresan D, Garber L (2016). Patient portals: an underused resource for improving patient engagement. Chest.

[64] Archer N, Fevrier-Thomas U, Lokker C, McKibbon KA, Straus SE (2011). Personal health records: a scoping review. J Am Med Inform Assoc.

[65] Giardina TD, Modi V, Parrish DE, Singh H (2015). The patient portal and abnormal test results: an exploratory study of patient experiences. Patient Exp J.

[66] Giardina TD, Baldwin J, Nystrom DT, Sittig DF, Singh H (2018). Patient perceptions of receiving test results via online portals: a mixed-methods study. J Am Med Inform Assoc.

[67] Geiss LS, Wang J, Cheng YJ, Thompson TJ, Barker L, Li Y, Albright AL, Gregg EW (2014). Prevalence and incidence trends for diagnosed diabetes among adults aged 20 to 79 years, United States, 1980-2012. J Am Med Assoc.

[68] Lyles CR, Harris LT, Jordan L, Grothaus L, Wehnes L, Reid RJ, Ralston JD (2012). Patient race/ethnicity and shared medical record use among diabetes patients. Med Care.

